# The Obesity Paradox in Cancer: a Review

**DOI:** 10.1007/s11912-016-0539-4

**Published:** 2016-07-30

**Authors:** Hannah Lennon, Matthew Sperrin, Ellena Badrick, Andrew G. Renehan

**Affiliations:** 1 Institute of Cancer Sciences, University of Manchester, Manchester, UK; 2 Farr Institute, MRC Health eResearch Centre (HeRC North), University of Manchester, Manchester, UK

**Keywords:** Body mass index, Excess weight, Adiposity, BMI, Cancer, Prognosis, Cancer survival, Mortality, Overweight, Obesity, Epidemiology

## Abstract

There is a common perception that excess adiposity, commonly approximated by body mass index (BMI), is associated with reduced cancer survival. A number of studies have emerged challenging this by demonstrating that overweight and early obese states are associated with improved survival. This finding is termed the “obesity paradox” and is well recognized in the cardio-metabolic literature but less so in oncology. Here, we summarize the epidemiological findings related to the obesity paradox in cancer. Our review highlights that many observations of the obesity paradox in cancer reflect methodological mechanisms including the crudeness of BMI as an obesity measure, confounding, detection bias, reverse causality, and a specific form of the selection bias, known as collider bias. It is imperative for the oncologist to interpret the observation of the obesity paradox against the above methodological framework and avoid the misinterpretation that being obese might be “good” or “protective” for cancer patients.

## Introduction

Excess body adiposity is a major global public health problem, with 67 % of the US, 63 % of the UK, and 64 % of Australia’s population being classified as overweight or obese, by body mass index (BMI) criteria, in 2014 [1]. A report from the World Cancer Research Fund (WCRF) [2], and a systematic review with standardized meta-analysis from one of the present authors [3], established, approximately a decade ago, that elevated BMI is associated with increased cancer incidence for several common adult cancer types. There are now ten established obesity-related cancers listed by the WCRF, including post-menopausal breast, endometrial, ovarian, advanced prostate, colorectal, renal, pancreatic, liver, and gallbladder cancers and esophageal adenocarcinoma. There is a common perception that, compared with normal-weight patients, elevated BMI is also associated with poorer prognosis after cancer diagnosis. This certainly is observed in systematic reviews of the literature among women with breast cancer [4] and forms a key rationale for weight management recommendations among cancer survivors, endorsed by clinical guidelines, for example, by the American Society of Clinical Oncology [5], with similar recommendations from the American Cancer Society [6] and European Society for Medical Oncology [7].

However, a number of isolated historic studies [8–10] and an emerging number of recent studies [11–15] have observed that among patients with cancer, elevated BMI is associated with improved survival compared with normal-weight patients. The surprising nature of this finding suggests the existence of an “obesity paradox”. This phenomenon is well described in the cardiovascular and metabolic literature [16–21] but less well appreciated in oncology. The repeated observation of the obesity paradox has spawned research that attempts to explain its occurrence. Posited explanations range from methodological (observed associations that contradict underlying causality due to confounding and bias) to clinical (seeking mechanistic explanations for obesity acting protectively in specific populations). In this review, we first explain what the obesity paradox is; summarize the current epidemiological findings for the association between overweight or obese status at cancer diagnosis and subsequent survival; review clinical and methodological explanations for the obesity paradox; and conclude with clinical implications and recommendations for further research.

## What Is the Obesity Paradox?

**Fig. 1:**
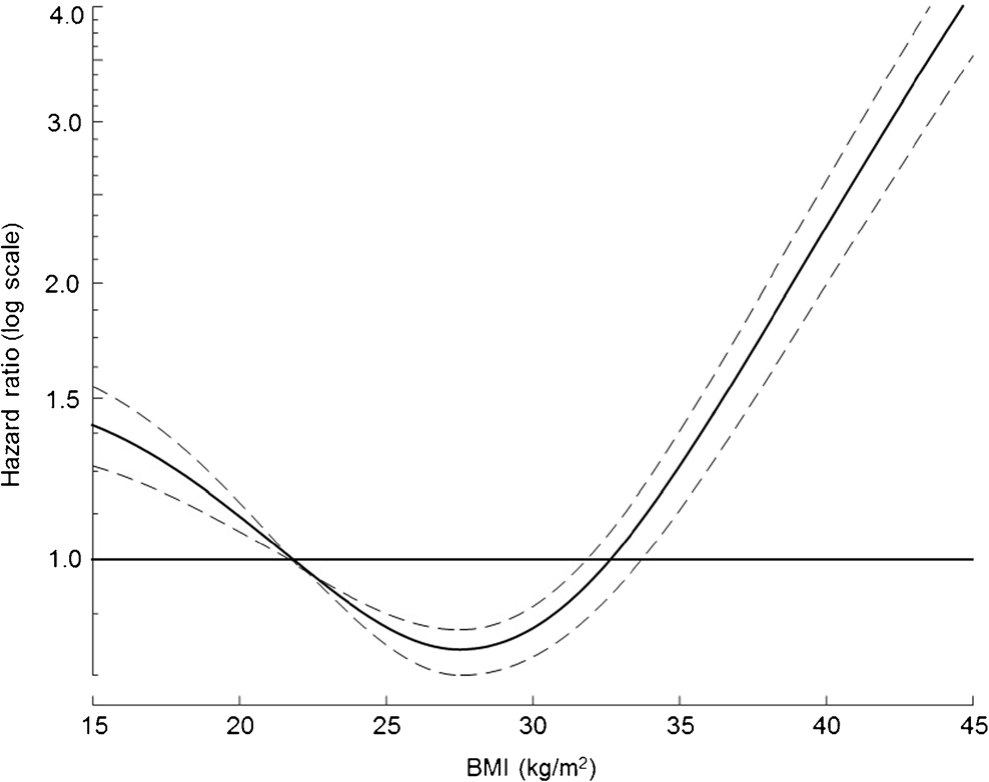
An illustration of the obesity paradox. The *vertical axis* represents hazard ratio of mortality (log scale), compared with the baseline BMI of 22.5 kg/m^2^. The *plot* represents a population in which the obesity paradox is observed, since the hazard ratio is below 1 in the overweight and obese range. The 95 % confidence intervals are shown with *dashed lines*

A BMI of 22.5 kg/m^2^ has been widely accepted as a mid-reference point for normal weight [22]. The obesity paradox occurs where the risk of outcome, typically mortality, is significantly reduced for BMI values above this referent, where an increased risk is expected. At very high BMI values, risk either returns to unity or is increased as illustrated in Fig. 1.

## Epidemiological Evidence

There have been mixed findings in incident cancer populations where there has been exploration for the obesity paradox, with the paradox being observed in some studies [8–11, 13–15, 23], but not in all [24–26]. Consequently, there have been attempts to unify the conflicting results in the literature with systematic reviews on adiposity and cancer survival [27–30] but with inconsistent summaries.

The obesity paradox has been observed in different cancer settings including, for example, in patients with colorectal cancer undergoing surgery [11]; patients with renal cancer undergoing surgery [10, 12]; patients with colorectal metastases undergoing liver resection [13]; elderly patients with acute myeloid leukemia [14]; and patients with lymphoma undergoing autologous hematopoietic cell transplantation [9]. The obesity paradox is not limited to non-metastatic disease and has been observed in a study of 4010 Taiwanese patients where the most common metastases were the lungs, liver, brain, and bone, requiring radiotherapy [15], and the hazard ratios decreased across BMI categories (overweight: HR 0.84 and obese: HR 0.67).

## General Points on Interpretation

Given the variations in study findings, there is a need to have an initial framework to interpret whether the obesity paradox is a true or artificial association. There are two broad principles to consider in the study characteristics: (i) when (in relation to cancer diagnosis) BMI was determined and (ii) the age of the participants under study.

When BMI was determined is relevant. The recent WCRF report on the effect of risk factors on survival among women with breast cancer added a very useful classification—namely, determination of BMI either at pre-, peri-, or post-diagnosis (the later typically 12 months after the initial treatment) of cancer [31]. From these, different patterns of associations emerge. In a meta-analysis of 29 studies evaluating the impact of BMI on survival in patients with colorectal cancer, Wu et al. [30] observed that increasing pre-diagnosis BMI prognosticated for a poor survival but that post-treatment overweight was associated with improved survival, i.e., the obesity paradox. Table 1 demonstrates that the obesity paradox can be illustrated in all three settings of pre- [32], peri- [15], and post-diagnosis [11] for different cancer types.

**Table 1:** Examples of studies demonstrating the obesity paradox in patients with cancer, where BMI was determined either pre-, peri-, or post-diagnosis of cancer

Study	Time of BMI determination	Cancer	Number and country (% male)	BMI categories kg/m^2^	Results	Adjusted for	Comment
Reichle et al. (2015) [32]	Pre-diagnosis	Prostate (*n* = 1958), colorectal (*n* = 1013), breast (*n* = 1410), gynecological (*n* = 572), upper digestive tract (*n* = 635), urogenital (*n* = 607), lymphatic (*n* = 508), others (*n* = 1970)	*n* = 8673 Austria (58 %)	<18.5 18.5–24.9 25.0–29.9 ≥30.0	1.28 (1.02–1.60) 1.00 (referent) 0.93 (0.87–0.99) 1.06 (0.97–1.15)	Age at cancer diagnosis, sex, smoking status, primary location, stage	
Tsang et al. (2016) [15]	Peri-diagnosis (date of receiving radiation therapy)	Distant metastases (bone, brain, others) with primary tumors (lung, breast, others)	*n* = 4010 Taiwan (55 %)	≤18.5 18.5–25.0 25.0–29.9 ≥30.0	1.41 (1.26–1.58) 1.00 (referent) 0.84 (0.78–0.91) 0.68 (0.57–0.81)	Age current, sex, performance status, primary tumor site, site of metastasis, multiple, onset of metastasis. EQD, chemotherapy, comorbidities, employment, alcohol, smoking, betel quid chewing, rural town	Alcohol, smoking, age, and comorbidities have a *P* value greater than 0.05
Schlesinger et al. (2014) [11]	Post-diagnosis (average 4 years after diagnosis)	Colorectal	*n* = 2143 (and *n* = 7565 in a meta-analysis) Germany (57 %)	25 ≤ 25.0–29.9 ≥30.0	2.12 (1.18–3.80) 1.00 (referent) 0.79 (0.71–0.89) 0.91 (0.80–1.04)	Age current, sex, alcohol, smoking status, tumor location, family history of CRC, metastases, other cancers (initially)	Prospective cohort study then meta-analysis with 7565 CRC patients

*BMI* body mass index, *EQD* equieffective dose (of radiotherapy), *CRC* colorectal cancer

Age is an additional attribute for consideration. For example, studies involving patients with leukemia are challenging to interpret due to the great age ranges of included individuals. Navarro and colleagues [33] showed that in over 4000 adults with acute myeloid leukemia under marrow transplantation, the obesity paradox was absent in young patients but present in those over age 60. Similar findings were noted by Brunner and colleagues, in a treatment cohort of adults with AML aged greater than 60 years [14].

## Explanations for the Obesity Paradox

Determining whether the obesity paradox is a causal phenomenon among patients with cancer is clinically relevant, as it informs weight management strategies among cancer survivors. There are many potential causes of the obesity paradox, and understanding these is central to clinical implications. These are grouped into two broad categories [16]: the first is methodological and reflects spurious or artificial associations; the second is clinical and potentially reflects true associations and is clinically useful.

### Methodological Explanations

#### BMI as an Inadequate Measure of Adiposity

BMI is commonly used as an approximation of general body adiposity in studies that have observed the obesity paradox. BMI is appealing as it is routinely measured in primary care and hospital settings and there are well-defined criteria for normal, overweight, and obese categories. However, BMI is a relatively crude measure of body adiposity and body composition and does not differentiate between lean mass and fat mass. In turn, body composition varies with age, sex, and ethnicity [22], such that there are currently no specific age-gender-ethnicity indices to define obesity in a standardized manner. Thus, for example, in a cancer population, overweight individuals (defined by BMI) might be younger with high muscle mass (compared with normal weight), explaining their better outcome compared with normal weight.

The paradox might not exist if alternate measures of body composition or adipose tissue were used. Thus, for example, we found no examples of studies in patients with cancer demonstrating the obesity paradox when anthropometric measures other than BMI or body composition indices were used. Alternate indices include measurements such as waist circumference, waist to hip ratio, skinfold, and body composition assessment techniques such as dual-energy X-ray absorptiometry, CT, and MRI, and quantify different body fat components such as subcutaneous adipose tissue (SAT) and visceral  adipose tissue (VAT) [34, 35]. Gonzalez and colleagues [36] recently explored this hypothesis and showed that the obesity paradox was present in 175 patients with various cancers (breast, gynecological, head and neck, lung, and gastrointestinal) when BMI was the exposure of interest but disappeared when obesity was defined using fat mass index and fat-free mass index.

#### Confounding

Confounding occurs when there are variables that are associated with both the outcome (death) and the exposure (obesity) and are not on the causal pathway between them. A common example is smoking, where BMI values are generally lower in current smokers than in never smokers. Other examples include deprivation, socioeconomic status, physical activity, and diet. It is difficult to adjust for all confounding factors, as many are unobserved. Measurement error may lead to incomplete removal of confounding, and to avoid this in the smoking context, some studies exclude smokers from their analysis [37], but usually, this is at the cost of disregarding a large proportion of the sample. Another approach would be to quantify exposure more accurately, for example, in terms of smoking duration and intensity using pack-years variables or cumulative lifetime exposure.

An example of confounding as a source of a spurious obesity paradox is illustrated in clinical treatment series reported by Hakimi and colleagues [12] in 2119 patients with renal cell carcinoma undergoing surgical resection at Memorial Sloan Kettering Cancer Center. Higher BMI was associated with reduced cancer-specific mortality in univariable analyses (*P* < 0.005), but this association was lost after adjusting for stage and grade (*P* > 0.10).

#### Selection Bias/Collider Stratification Bias

The obesity paradox might be due to a specific form of selection bias, known as the collider stratification bias, caused during the statistical analysis due to conditioning on a subpopulation selected based on a collider variable. In turn, a collider variable is one with at least two causes common to the risk of the variable and the outcome of interest. For example, cancer incidence is a collider variable because it is “caused” by both obesity and other risk factors (e.g., smoking). There is a well-recognized inverse relationship between BMI and smoking. Thus, cancer patients who are not obese are more likely to have other risk factors, such as smoking, and in the analyzed subpopulation, an inverse association is artificially generated (or strengthened) between obesity and the other risk factors. Additionally, Banack and Kaufman [38••] demonstrate how confounding due to smoking is increased in the presence of collider stratification bias. In a contrary direction, Sperrin et al. [39••], using an equation-derived approach within a counterfactual framework, show that the biases attributable to collider stratification are small and cannot explain the large paradoxical relationships seen in epidemiological studies.

#### Detection Bias

A further dimension is detection bias. This is the co-occurrence of two diagnoses together. Thus, for example, being overweight and obese is associated with the development of diabetes and cardiovascular disease. Where patients present with new diagnoses of these conditions, they undergo several investigations, which in turn detect incidental diseases including silent cancers—a form of “opportunistic surveillance”. This overestimation of the occurrence of cancer diagnosis concurrent with a new diagnosis of diabetes is well recognized [40]. These silent cancers might have a low-stage disease with generally good prognosis and account for the obesity paradox in overweight and obese patients. One approach to minimize detection bias is to adjust for tumor stage at presentation or alternatively where the date of diagnosis of, say, diabetes is known, use a washout of 2 years to “bypass” the detection bias.

#### Reverse Causality

Reverse causality refers to the phenomenon that some normal-weight patients may have previously been obese but lost weight due to illness, here cancer. Cancer is known to cause weight loss by loss of appetite or increased metabolic demands. The extent of weight loss correlates with initial BMI and occurs in patients with early-stage as well as late-stage tumors [41]. The potential causal link of reverse causality with the obesity paradox has been shown in several examples. Thus, Gelber and colleagues [42] evaluated the relationship between BMI and mortality in 99,253 male physicians in the Physicians’ Health Study and initially showed a U-shaped association with all-cause mortality, which converted to a linear relationship in their optimal model excluding men who died within 2 years of initial assessment. Similarly, Tseng [43] evaluated a nationally representative cohort of 89,056 Taiwanese patients with type 2 diabetes, matched with the National Death Certificate Database, and on initial analysis found that BMI was inversely associated with mortality from all-cause, cancer, and diabetes complications, but after excluding patients with a follow-up duration less than 2 years, BMI categories were not significantly prognostic for cancer-related mortality, suggesting a bias induced by cancer-induced weight loss.

Reverse causality is better explored and minimized by using longitudinal data to obtain a description of the patient’s usual weight and its trajectory up to cancer diagnosis. Weight histories (repeated weight measurements) give a useful dimension of obesity exposure, but there is a paucity of such data. Research to define obesity-equivalence of pack-years will be informative. A simpler approach when weight histories are not available is to include an individual’s maximum lifetime BMI and is robust to confounding by illness-induced weight loss. Stokes and Preston [37] have illustrated this approach demonstrating a reduction in biases when including maximum lifetime BMI into their models. This approach is similar to evaluating weight change, for example, the rate of change and size of variability over time [28, 44].

### Clinical Explanations

#### Tumor Biology Is Less Aggressive

There are some examples where tumors among obese patients have less aggressive characteristics compared with those among normal-weight patients. In obese women with endometrial cancer, there is a predominance of good prognosis type 1 tumors compared with poor prognosis type 2 endometrial cancer. Tumors are molecularly heterogeneous, and it is speculated that obesity is associated with less aggressive biological subtypes. For renal carcinoma, obesity is associated with more indolent molecular variants (for example, reduced fatty acid synthase, *FASN*, gene expression) [12], while in contrast, for ovarian cancer, elevated BMI is associated with good prognosis cancers (low-grade serous and endometrioid), but within this histological type subpopulation, there is a linear positive association between BMI and mortality, which is absent in high-grade serous ovarian cancers [45].

#### Tumors Respond Better in Obese Patients

The overweight and obese state might influence treatment outcomes, both in terms of how the tumor (changed for consistency) behaves to treatment and in terms of the differential pharmacokinetics of cancer treatment regimens. For example, high-intra-abdominal fat volume predicts for greater doxorubicin exposure and hematologic toxicities in women with breast cancer compared with body surface area [46]. Similarly, overweight and obese patients might be differentially allocated to less radical cancer surgery, though Gurunathan and Myles [47] point out the limitations of BMI as a predictor of peri-operative complication risk and indicate that mildly obese and overweight patients outperform normal-weight patients after many types of surgeries.

A specific mention is worthwhile for the complex inter-relationships between adjuvant chemotherapy (after curative resection) and the overweight/obese state. There is a well-recognized clinical practice among many oncologists to dose cap chemotherapy in obese patients with body surface area (BSA) greater than 2.0, and together with differential allocation and differential adherence to adjuvant chemotherapy, obese patients may simply have poorer outcomes compared with normal-weight patients because they are sub-optimally treated. This is illustrated by Sinicrope and colleagues [48], who examined the prognostic impact of BMI in 25,291 patients with stage II and III colon carcinoma within the Adjuvant Colon Cancer Endpoints (ACCENT) database, a consortium of randomized trials of 5-fluorouracil-based adjuvant chemotherapy. With disease-free survival (DFS) as a key outcome measure, compared with normal-weight patients, they showed a significant reduction in DFS limited to men with class 2 and 3 obesity (BMI ≥35.0 kg/m^2^) but an improved survival for overweight and class 1 obese (BMI 25.0 to 34.9 kg/m^2^) men, i.e., the obesity paradox. There were different dose-capping practices among the trials, and there was lack of data on chemotherapy adherence by BMI status, such that the study was unable to conclude whether or not these confounders contributed to the differential impact of BMI states on survival.

#### Energy Reserve or Hibernation Hypothesis

A third hypothesis is that excess adipose tissue serves as a nutrient reserve and confers a survival advantage in times of stress, such as anti-cancer treatment. This is akin to the hibernation theories in evolutional biology whereby species store up energy in anticipation of harsh times ahead. On a parallel note, it remains unclear if obesity drives cancer progression, whether it is due to excess adiposity or the energy imbalance [49].

## Conclusions and Future Directions

This review has highlighted the mixed findings in studies evaluating the obesity paradox in cancer populations. In terms of interpreting these studies, and designing future studies on this topic, there is a need to apply a methodological framework to determine whether the obesity paradox is a true or spurious relationship for a given setting. If a framework is not used, mistaken interpretations can be reached. Thus, in the cardiovascular literature, some commentators have concluded that the obesity paradox is a true causal association arguing that the optimum body weight is above the normal BMI range in individuals with some chronic diseases [17].

First, where the primary interest is the effect of obesity on survival, it is preferred to incorporate as much information regarding the patients’ weight history, i.e., consider the patient’s BMI trajectory throughout a long period of time or ideally through the whole life-course. Variations on this include modeling BMI at pre-, peri-, and post-diagnosis of cancer. An alternative is to use maximum lifetime weight and weight variability measures.

Second, it is important to work within datasets with richness for potential confounding. The following are potential effect modifiers or confounders of the relationship between BMI and survival but are not always captured: smoking, hormonal replacement therapy, and ethnicity. Cancers diagnosed through screening programs have better prognosis than non-screened cancer, and in turn, obesity tends to be associated with lower uptake rates in cancer screening.

Third, conflicting findings may be partly explained by heterogeneity within cancer types, the timing of when BMI was determined, unmeasured confounders, and statistical biases. To further understand the observed associations, directions for future research include (i) improving the “subtyping” of cancer by better recording of staging, tumor type; (ii) improving data linkage so BMI, adiposity measures, and confounding variables can readily be extracted from records and incorporated; and (iii) further research into the gender-specific links of the effect of obesity and overweight on survival in cancer populations.

It is imperative for the oncologist to interpret the observation of the obesity paradox against the above methodological framework and avoid the misinterpretation that being obese might be “good” or “protective” for cancer patients. ‘First, do no harm’.
